# Intraoral lipoma: an atypical case

**DOI:** 10.1590/S1808-86942011000500024

**Published:** 2015-10-22

**Authors:** Luiz Carlos Oliveira dos Santos, Stela Maris Wanderley Rocha, Camila Nunes Carvalho, Ericka Porangaba Accioly de Oliveira, Davi Felipe Costa Neves

**Affiliations:** 1 Doctoral degree in stomatology. Adjunct professor of stomatology, UFAL; 2 Master's degree in oral and maxillofacial surgery. Assistant professor of oral and maxillofacial surgery; 3 Dentistry student, UFAL; 4 Dentistry student, UFAL; 5 Undergraduate course in dentistry, dental surgeon

**Keywords:** lipoma, mouth, mouth neoplasms

## INTRODUCTION

A lipoma in the mouth is an asymptomatic slowly growing benign tumor of mesenchymal origin consisting of fat.^1^ It may present in various forms, as a sessile or pediculated and single or lobulated tumor of variable sizes although mostly below 3 cm diameter, and generally surrounded by a fibrous capsule.

These lesions are soft, and rarely develop in the mouth; in this site, 50% of these tumors may be found in the jugal mucosa or the vestibule^1^.

The etiology of lipomas is uncertain; some authors have suggested endocrine, traumatic, and hereditary causes^1^.

The diagnosis is made by pathology of an incisional or excisional specimen. An important feature is that the tumor tends to float when placed in a 10% formaldehyde solution^2^. Treatment consists of conservative surgical removal of the lipoma; recurrences are rare.

The purpose of this study was to report a case of a patient with a large oral lipoma; treatment consisted of surgical excision. The study includes a case report and a review of the literature.

## CASE REPORT

**Figure 1:**
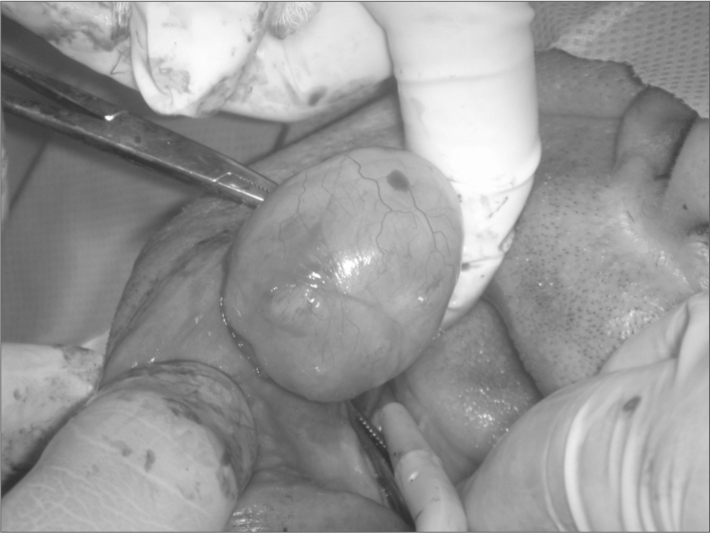
Lipoma - Resection of tumor.

J.B.V, a 58-year-old white male patient from the city of Maceió, AL, was referred to the stomatology unit of a dentistry school in the state of Alagoas; he presented a nodule in the right region of the mouth. The patient informed that the tumor had grown within the last six months, and that he was unable to fit a lower dental appliance, which affected chewing and speech; he reported no pain. A large intraoral smooth well-defined sessile nodule of similar color to the surrounding mucosa was observed in the jugal mucosa, a little above the alveolar ridge and the mentonian foramen; it measured about 5 cm on its longer axis. Radiography revealed no bone involvement. An excision biopsy was carried out (Figure 1). The specimen was placed in 10% formaldehyde, where it floated, suggesting fat content. It was sent to pathology, which confirmed that it was a lipoma. The patient is currently being monitored and so far no recurrence has occurred.

## DISCUSSION

Lipomas of the mouth are benign tumors; they grow slowly, do not infiltrate other tissues, do not ulcerate, and are painless. They are relatively rare in the mouth and the maxilla-facial region^3^,^4^. The case above fits into these criteria and other reports in the literature; it is atypical in its size (5.0 cm).

According to the literature, mouth lipomas are distributed evenly between sexes; most of these patients are aged over 40 years^5^. The case is similar to other published reports, as the patient was aged 57 years.

The diagnosis is clinical and histopathological. The differential diagnosis includes ranulae, epidermoid cysts, pleomorphic adenomas, and fibromas.^1^

The treatment, irrespective of histological variation, is surgical^3^, which was the therapy of choice in the present case.

## FINAL COMMENTS

A conservative approach was chosen for the present case; which has so far been effective.

It is therefore important to diagnose the lesion correctly in the physical examination and histopathology to establish the prognosis. Healthcare professionals need to understand this disease to treat it adequately.

## References

[1] Capelari MM, Marzola C, Toledo Filho JL, Azenha MR, Pereira LC, Alonso de Moura L. (2008). Extenso lipoma da cavidade bucal, associado ao plexo vásculo-nervoso mentual.. Rev ATO..

[2] Fregnani ER, Pires FR, Falzoni R, Lopes MA, Vargas PA. (2003). Lipomas of the oral cavity: clinical findings, histological classification and proliferative activity of 46 cases.. Int J Oral Maxillofac Surg..

[3] Aniballi S, Cristalli MP, La Monaca G, Giannone N, Testa NF, Lo Russo L (2009). Lipoma in the soft tissues of the floor of the mouth: A case report.. Open Otorhinolaryngol J..

[4] Esmeili T, Lozada-Nur F, Epstein L. (2005). Common benign oral soft tissue masses.. Dent Clin North Am..

[5] Furlong MA, Fanburg-Smith JC, Childers EL. (2004). Lipoma of the oral and maxillofacial region: Site and subclassification of 125 cases.. Oral Surg Oral Med Oral Pathol Oral Radiol Endod..

